# Identification alpha-2-HS-glycoprotein precursor and tubulin beta chain as serology diagnosis biomarker of colorectal cancer

**DOI:** 10.1186/1746-1596-9-53

**Published:** 2014-03-12

**Authors:** Nai-Jun Fan, Rui Kang, Xue-Yan Ge, Ming Li, Yan Liu, Hong-Mei Chen, Chun-Fang Gao

**Affiliations:** 1 Institute of Anal-colorectal Surgery, No.150 Central Hospital of PLA, Luoyang, RP China; 2 The General Hospital of Jinan Military Region, Jinan, China; 3 Respiration Department, No.150 Central Hospital of PLA, Luoyang, RP China; 4 Neuro- Surgery Department, No.150 Central Hospital of PLA, Luoyang, RP China; 5 Clinical Laboratory, The First People’s Hospital of the City of Luoyang, Luoyang, RP China

**Keywords:** Colorectal neoplasms, Diagnosis, Biological markers, Proteomics, Alpha-2-HS-glycoprotein, Tubulin beta chain

## Abstract

**Background:**

Colorectal cancer (CRC) remains a major worldwide cause of cancer-related morbidity and mortality largely due to the insidious onset of the disease. The current clinical procedures utilized for disease diagnosis are invasive, unpleasant, and inconvenient. Hence, the need for simple blood tests that could be used for the early detection is crucial for its ultimate control and prevention.

**Methods:**

The present work is a case–control study focused on proteomic analysis of serum of healthy volunteers and CRC patients by the ClinProt profiling technology based on mass spectrometry. This approach allowed to identifying a pattern of proteins/peptides able to differentiate the studied populations. Moreover, some of peptides differentially expressed in the serum of patients as compared to healthy volunteers were identified by LTQ Orbitrap XL.

**Results:**

A Quick Classifier Algorithm was used to construct the peptidome patterns (m/z 1208, 1467, 1505, 1618, 1656 and 4215) for the identification of CRC from healthy volunteers with accuracy close to 100% (>CEA, *P* < 0.05). Peaks at m/z 1505 and 1618 were identified as alpha-2-HS-glycoprotein precursor and tubulin beta chain, respectively.

**Conclusions:**

Alpha-2-HS-glycoprotein precursor and tubulin beta chain could be involved in the pathogenesis of CRC and perform as potential serology diagnosis biomarker.

**Virtual slides:**

The virtual slide(s) for this article can be found here: http://www.diagnosticpathology.diagnomx.eu/vs/4796578761089186.

## Introduction

Colorectal cancer (CRC) is the third most commonly diagnosed cancer in males and the second in females, with over 1.2 million new cancer cases and 0.61 million deaths estimated to have occurred in 2008. This is due to the insidious onset of the disease largely [1]. More than 60% of patients with CRC are either locally or distantly invasive at diagnosis, restricting treatment options and reducing survival rates, whereas the 5-year survival rate is extremely favorable if detected at an early stage and treated early, while the tumors were still localized [2-5]. However, the early diagnosis rate is still comparatively low as the current clinical procedures utilized for CRC diagnosis are either invasive, unpleasant, inconvenient or low sensitivity [6,7]. Hence, the need for simple blood tests that could be used for the early detection, which emerging with potential to improve screening effectiveness and userfriendliness, is crucial for its ultimate control and prevention.

Biomarkers are important tools for cancer detection and monitoring. They serve as hallmarks for the physiological status of a cell at a given time and change during the disease process, including CRC [8-10]. Many existing cancer biomarkers are glycoproteins, such as carcinoembryonic antigen (CEA) in CRC. Despite of remaining the most widely used serum biomarker, CEA is precluded its use for the early detection of CRC due to poor sensitivity and specificity [11]. The emerging field of clinical proteomics is especially well suited to the discovery and implementation of potential biomarkers, as body fluids are an cellular and protein-rich information reservoir that contains traces of what the blood has encountered during its circulation through the body [12]. Serum proteome analysis has the potential to facilitate disease diagnosis and therapeutic monitoring, because serum is more easily accessible and widely collected sample, which contains >10,000 different proteins and peptides [13-17]. A novel technology platform, called ClinProt (Bruker Daltonics, Ettlingen, Germany), comprising a magnetic bead (MB) based sample separation, matrix-assisted laser desorption/ ionization time of flight mass spectrometry (MALDI-TOF MS) for protein/peptide profiling acquisition, and a bioinformatics package for inspection and comparison of data sets to create “disease-specific” protein/peptide patterns models, could serve as a powerful tool for the diagnosis of cancer [15,16,18-23]. Previously, our group quantified 61 dysregulation proteins/peptides in CRC, including 49 up-regulated and 12 down-regulated proteins/peptides using this technology platform [24]. However, the dysregulation proteins/peptides were not identified. LTQ Orbitrap XL MS/MS (Michrom Bioresources, Auburn, USA) shows great potential for protein identification [25]. Therefore, we applied the combination of MB, MALDI-TOF MS and LTQ Orbitrap XL MS/MS for globally analyze and identification of the serum biomarkers associating with CRC.

## Materials and methods

### Reagents and instruments

The AutoFlex III MALDI-TOF MS, MTP 384 target plate polished steel, α-cyano-hydroxycinnamic (CHCA), MB-WCX kit and peptide calibration standard were purchased from Bruker Daltonics (Leipzig, Germany). Trifluoroacetic acid (TFA) was purchased from Alfa Aesar (Ward Hill, MA, USA). Acetonitrile (ACN) was acquired from Sigma (St. Louis, MO, USA). Diagnostic Kit of Carcinoembryonic antigen (CEA) (ELISA) was acquired from Roche Diagnostics GmbH (Sandhofer Strasse, Germany). Nano Aquity UPLC was acquired from Waters Corporation (Milford, USA) and LTQ Orbitrap XL MS/MS was acquired from Michrom Bioresources (Auburn, USA).

### Patients, health volunteers and sample collection

Valid records of No.150 Central Hospital of the People’s Liberation Army (PLA) Cancer Center were searched for patients with a histological diagnosis of CRC whose serum samples had been obtained from January of 2011 to December of 2012. Health volunteers come from the healthy physical examination in No.150 Central Hospital of PLA January of 2011 to December of 2012. Seventy health volunteers (blood donor volunteers) and 72 CRC patients were enrolled with the permission of the Local Ethical Commission, and blood was collected after informed consent from the patients. Enteroscopy were performed in all health subjects to exclude the presence of incidental colon and rectum mass. CRC patients underwent clinical staging, surgical excision of the lesion, and were followed up. Pathologic samples were classified according to the 2004 tumor-node-metastasis (TNM) stage classification [26]. The clinical characteristics of CRC patients were shown in Table 1.

**Table 1:** Clinical characteristics of colorectal cancer patients recruited in model construction group and external validation group

**Clinical characteristics**	**Model construction group**	**Evaluation group**	***P*-value**
	**(n = 36)**	**(n = 36)**	
Gender: male/female	19/17	19/17	1.000^§^
Age (years, $\bar{X}\pm \mathrm{SD}$)	63.58 ± 10.02	61.19 ± 11.48	0.875^#^
TNM stage			0.629
	6	5	
	17	16	
	12	13	
	1	2	
Tumor localization			0.629^§^
Colon	15	13	
Rectum	21	23	

^§^Pearson Chi-Square test.

^#^Independent-Sample T Test.

Serum samples were prepared by collecting blood in a vacuum tube and allowing it to clot for 30 minutes at room temperature. About 1 mL of serum was obtained after centrifugation at 2000 rpm for 10 minutes, and it was stored in small aliquots at −80°C until analysis.

### Study design

The data set including 70 healthy volunteers and 72 CRC patients was randomly split into model construction group and external validation group. The model construction group (including 35 health volunteers and 36 CRC patients) was used for the identification of signals related to proteins/peptides expressed differentially in CRC patients compared with health volunteers and diagnosis patterns recognition. The external validation group (including 35 health volunteers and 36 CRC patients) was used for the independent validation of the diagnosis patterns blindly. The accuracy of the peptide model was compared with that of CEA.

The gender ratio (male/female) of health volunteers and CRC patients was 1.12 and 1.18, respectively. The mean age (years) of health volunteers and CRC patients was 58.94 ± 8.32 and 62.43 ± 9.37, respectively. The difference of age and gender of health volunteers in model construction group and external validation group were not significantly. No significant differences were observed for TNM stage of CRC between model construction group and external evaluation group (Table 1).

### Sample purification

We used MB-WCX for proteins/peptides separation of samples following the standard protocol by the manufacturer [27]. Briefly, the sera and ClinProt Kit were left at room temperature for 2 h for serum protein fractionation, 10 μL of WCX-MB binding solution and 10 μL of WCX-beads were combined in a 0.5 mL microfuge tube after thoroughly vortexing both reagents. Then, 5 μL of serum sample was added to the microfuge tube and mixed by pipetting up and down. The samples were then were then placed in a magnetic bead separator (MBS) where the beads were pulled to the side by magnetic force, allowing for the supernatant to be removed and discarded. After three washes with MB-WCX washing solution, the supernatant was removed and the beads remained in place. 5 μL of WCX-MB elution buffer was added to disperse beads in tubes by pipetting up and down. Then the beads were pulled to the side and a fraction of the eluate was transferred to another tube. 5 μL WCX-MB stabilization solutions were added to the collected supernatant, mixing intensively by pipetting up and down, the mixture was then ready for spotting onto MALDI-TOF MS targets and measurement. Finally, prior to the MALDI-TOF MS analysis, we prepared targets by spotting 1 μL of the proteome fraction on the polished steel target (Bruker Daltonics ). After air drying, 1 μL of 3 mg/mL CHCA in 50% ACN and 50% Milli-Q with 2% TFA was applied onto each spot, and the target was air dried again (co-crystallization). The peptide calibration standard (1 pmol/μL peptide mixture) was applied for calibrating the machine.

### Mass spectrometry analysis

For proteome analysis, we used a linear Autoflex III MALDI-TOF-MS with the following setting: ion source 1, 20.00 kV; ion source 2, 18.60 kV; lens, 6.60 kV; pulsed ion extraction, 120 ns; Ionization was achieved by irradiation with a crystal laser operating at 200.0Hz. For matrix suppression, we used a high gating factor with signal suppression up to 600 Da. Mass spectrum were detected using linear positive mode. Mass calibration was performed with the calibration mixture of peptides and proteins in the mass range of 1 000–20 000 Da. We measured three MALDI preparations (MALDI spots) for each MB fraction. For each MALDI spot, 1600 spectrum were acquired (200 laser shots at 8 different spot positions). Spectrum were collected automatically using the Autoflex Analysis software (Bruker Daltonik) for fuzzy controlled adjustment of critical instrument settings to generate raw data of optimized quality.

The criteria for protein mass peak detection (m/z) were as follows: signal-to-noise ratio (S/N) >5, a 2-Da peak width filter, and a maximum peak number of 200. The intensities of the peaks of interest were normalized with the peak intensity of an ACTH internal standard. More than 10% of the molecular weight was sieved in simultaneous samples, with the discrepancy of identical spinnacle in different samples <0.3% after removal of the initial data noise.

### Bioinformatics and statistical analysis

The ClinProt Tools software 2.2 (Bruker Daltonik) was used for analysis of all serum sample data derived from either patients or normal controls. Data analysis began with raw data pretreatment, including baseline subtraction of spectrum, normalization of a set of spectrum, internal peak alignment using prominent peaks, and a peak picking procedure. The pretreated data were then used for visualization and statistical analysis in ClinProt Tools. Statistically significant different quantity of peptides was determined by means of Wilcoxon test. The significance was set at p < 0.05. Class prediction model was set up by GA. A classify proteins/peptides patterns was constructed. To determine the accuracy of the class prediction, firstly, a cross-validation was implemented. Twenty percent of model construction group were randomly selected sample as a test set, and the rest samples were taken as a training set in the class predictor algorithm. Secondly, designed as double blind test, the samples of external validation group were classified by the classify proteins/peptides patterns constructed by GA.

### Identification of protein markers

Selected proteins/peptides were further purified using Nano Aquity UPLC C18 beads and serially eluted with 5% and 95% acetonitrile. These proteins/peptides were identified directly via LTQ Orbitrap XL (Michrom Bioresources, Auburn, USA) analysis in order to obtain the peptide sequences. For the Nano Ion Source, spray voltage was 1.8 kV, MS scan time was 60 min, and the scanning range was 400-2000 m/z. Obitrap was used for the first scan (MS), with resolution of 100000 and LTQ was used for CID and the second scan (MS/MS). The 10 strongest ion intensities in the MS spectrum were selected as the parent ion for the MS/MS (single charge exclusion, not as a parent ion). The obtained chromatograms were analyzed with Bioworks Browser 3.3.1 SP1 and the resulting mass lists were used for database search using Sequest™ (IPI Human (3.45)).

### Detection of CEA

The serum CEA of 36 CRC and 35 health volunteers included in external validation group was detected using an electrochemiluminescent immunoassay method following the standard protocol by the manufacturer (The methods were omitted). The sample was diagnosed as CRC (CEA ≥ 5 ng/ml), otherwise diagnosed as health volunteers (CEA < 5 ng/ml).

### Statistical methods, evaluation of assay precision

We analyzed each spectrum obtained from MALDI-TOF MS with AutoflexAnalysis and ClinProt TM software (Bruker Daltonics), the former to detect the peak intensities of interest and the latter to compile the peaks across the spectrum obtained from all samples. This allowed differentiation between the cancer and healthy volunteers’ samples. To evaluate the precision of the assay, we determined within- and between-run variations by use of multiple analyses of bead fractionation and MS for 2 plasma samples. For within- and between-run variation, we examined 3 peaks with various intensities. We determined within-run imprecision by evaluating the CVs for each sample, using 8 assays within a run, then determined between-run imprecision by performing 8 different assays over a period of 7 days. SPSS16.0 was used for analysis of the clinical characteristics of volunteers using χ2 test or *t* test. The significance was set at *P* < 0.05. Also, SPSS 16.0 was used to compare the diagnosis accuracy of the proteins/peptides models and CEA.

## Results

### System stability and experimental reproducibility were ensured through the use of 3 peaks with different molecular masses of standard serum

For the reproducibility of the protein profiling, within- and between-run reproducibility of 2 samples were determined with the WCX-MB fractionation and MALDI-TOF MS analysis. In each profile, 3 peaks with different molecular masses were selected to evaluate the precision of the assay. Despite varying proteins/peptides masses and spectrum intensities, the peak CVs were all <5% in the within-run and <10% in the between-run assays. These values were consistent with the reproducibility data for the Protein Biology System reported by the manufacturer (Bruker Daltonik).

### Differentiation of proteins/peptides selected out between healthy volunteers and CRC

**Figure 1:**
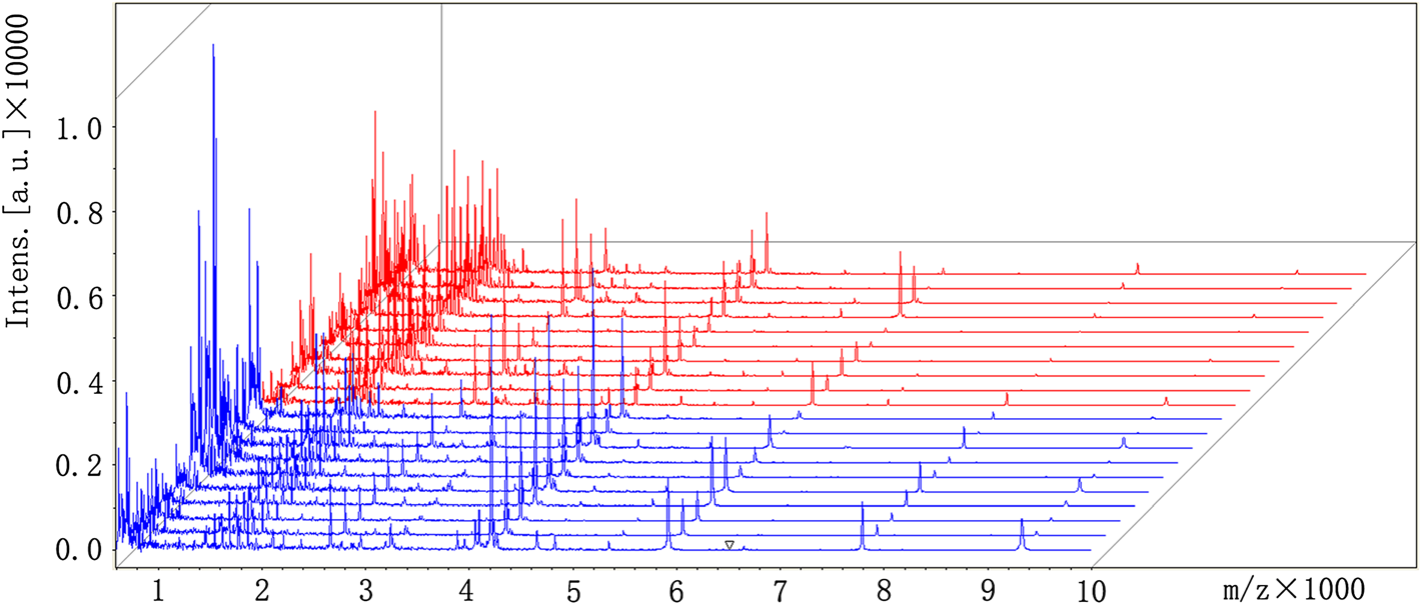
**View of the aligned mass spectrum of the serum protein profile of model construction group obtained by MALDI-TOF after purification with WCX magnetic beads.** Red represent 10 colorectal cancer patients, blue represent 10 healthy volunteers.

All healthy volunteers and CRC patients’ sera proteins/peptides profiles were analyzed using a new high-resolution MALDI-TOF MS coupled with bead fractionation. Samples were randomly distributed during processing and analysis. A total of 71 distinct m/z values were resolved in the 600–20000 Da range (Figure 1). Differences in peak positions and intensities were observed and later used to statistically analyze the spectrum. ClinprotTools ver 2.2 (Bruker Daltonic) was used for peak detection. Twenty four proteins/peptides (including 9 up-regulated and 15 down-regulated peptides) displayed significant statistical significance (P < 0.05) according to a Wilcoxon test between healthy volunteers and CRC patients groups. These data are shown in Table 2. Quantity analysis of these dysregulated proteins/peptides in CRCs showed that the expression levels were not correlated with the clinical characters of CRC, such as TNM stages, age, gender, et al.

**Table 2:** Statistics of the 24 dysregulated proteins in colorectal cancer compared with health individuals

**Mass**	**Colorectal cancer **^**▲**^	**Health volunteer **^**▲**^	**Regulation in colorectal cancer**	**P-value**^**★**^
	**(Average ± SD)**	**(Average ± SD)**		
1618.52※	64.84 ± 37	20.91 ± 17.59	Up-regulation	0.001
1467.44※	52 ± 36.99	12.41 ± 6.73	Up-regulation	0.000
4214.69※	45.08 ± 24.84	100.83 ± 26.97	Down-regulation	0.000
798.20	41.9 ± 26.18	17.04 ± 6.00	Up-regulation	0.004
825.73	25.51 ± 16.96	11.25 ± 3.07	Up-regulation	0.007
651.55	16.05 ± 15.27	30.14 ± 14.65	Down-regulation	0.007
1656.22※	13.77 ± 7.10	4.69 ± 2.63	Up-regulation	0.000
667.29	12.34 ± 9.65	19.76 ± 8.55	Down-regulation	0.028
1505.47※	10.81 ± 5.89	3.86 ± 1.18	Up-regulation	0.000
1521.72	10.49 ± 4.50	5.20 ± 2.38	Up-regulation	0.002
1208.30※	10.39 ± 4.48	4.27 ± 1.05	Up-regulation	0.001
1867.23	10.00 ± 6.35	17.64 ± 7.03	Down-regulation	0.018
1585.25	9.64 ± 3.93	5.51 ± 2.48	Up-regulation	0.004
4096.50	7.39 ± 4.77	14.26 ± 3.80	Down-regulation	0.005
1780.03	6.90 ± 5.09	11.21 ± 5.26	Down-regulation	0.019
7791.73	6.43 ± 6.97	13.01 ± 3.03	Down-regulation	0.014
4058.71	3.59 ± 2.73	15.02 ± 6.18	Down-regulation	0.000
4198.23	3.53 ± 2.77	7.44 ± 2.62	Down-regulation	0.003
4271.61	2.04 ± 1.71	4.64 ± 1.51	Down-regulation	0.000
4174.45	1.53 ± 1.25	2.63 ± 1.07	Down-regulation	0.021
4177.42	1.50 ± 1.25	2.51 ± 1.13	Down-regulation	0.026
4079.82	1.45 ± 1.04	2.49 ± 0.91	Down-regulation	0.014
4076.90	1.43 ± 1.04	2.57 ± 0.87	Down-regulation	0.007
3939.53	1.08 ± 0.51	1.88 ± 0.68	Down-regulation	0.005

※The peptide selected for model construction.

^▲^ Peak area.

★P value calculated with the Wilcoxon test; values lower than 0.05 suggest statistical relevance.

### Establishment and validation of predicting model

**Figure 2:**
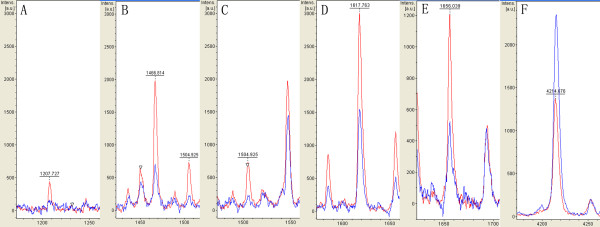
Zoom of the mass range for the six proteins (m/z 1208 (A), 1467 (B), 1505 (C), 1618 (D), 1656 (E) and 4215 (F), MALDI-TOF linear mode) used in the cluster to differentiate colorectal cancer (red) from healthy volunteers (blue).

**Figure 3:**
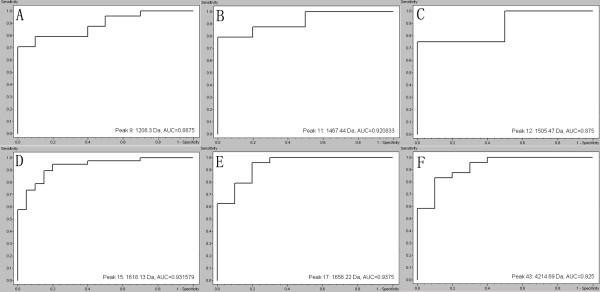
**Receiver operating characteristic curve of the six proteins (m/z 1208 (A), 1467 (B), 1505 (C), 1618 (D), 1656 (E) and 4215 (F)) selected for the diagnostic model.** AUC, Areas under the receiver operating characteristic curve.

**Figure 4:**
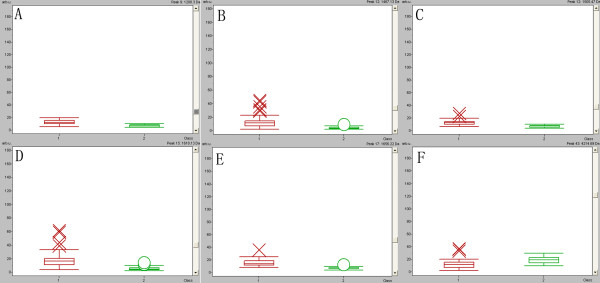
**Box-and-whiskers plot calculated from the areas of the six proteins (m/z 1208 (A), 1467 (B), 1505 (C), 1618 (D), 1656 (E) and 4215 (F)) selected for the diagnostic model.** Red represents colorectal cancer, Green represents healthy volunteers (*P* < 0.01 versus ctrl).

**Figure 5:**
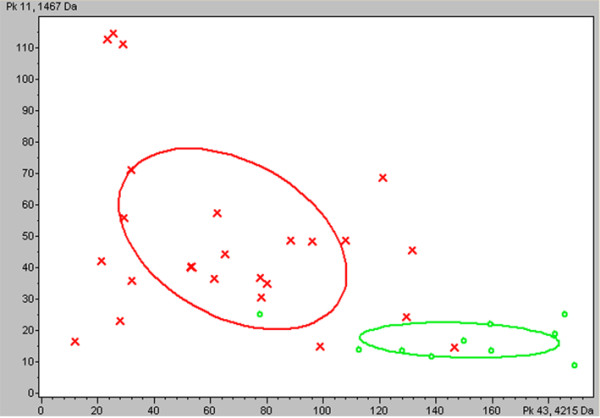
**Two dimensional peak distribution view of the two peaks (m/z 1467 and 4215).** The peak area and the m/z values are indicated on the x- and y-axes. The ellipses represent the standard deviation of the class average of the peak areas/intensities. Red represents colorectal cancer patients and green represents healthy volunteers.

Classification models were developed to discriminate CRC from health volunteers. A GA in ClinProt was trained with the detected peaks from the discovery set to generate cross-validated classification models. Among the differentially expressed proteins/peptides, six (m/z 1208, 1467, 1505, 1618, 1656 and 4215) were chosen by the GA to build up a possible diagnostic cluster of signals. Regions of the mass spectrum obtained at about 800 resolving power measured are reported in Figure 2. The diagnostic capability of each peak determined by ROC curve is reported in Figure 3. Moreover areas of these peaks in the spectrum of CRC and healthy volunteers were statistically different from those of the healthy volunteers (Figure 4). Combination of the six peaks was provided as the best predicting model, achieving a recognition capacity of 98.25% (a sensitivity of 96.55%and a specificity of 100%), with 20% of randomly selected data points omitted in the cross validation step. The accuracy of the models was verified with the validation set data, consisting of the 20% omitted samples. All the samples were correctly classified by the GA model (a sensitivity and a specificity of 100%). Combination of two of these signals at m/z 1505 and 1618 differentiated the two populations (Figure 5).

To verify the accuracy of the established GA classification model, we introduced another group of samples (not used in model construction), which consisted of 36 CRC and 35 health volunteers. As a result, the model correctly classified 94.44% (34/36) of CRC (sensitivity) and 94.29% (33/35) of health volunteers (specificity), which surpassed that of CEA (a specificity of 52.78% (19/36), and a sensitivity of 48.57% (17/35)).

### Identification of markers

**Figure 6:**
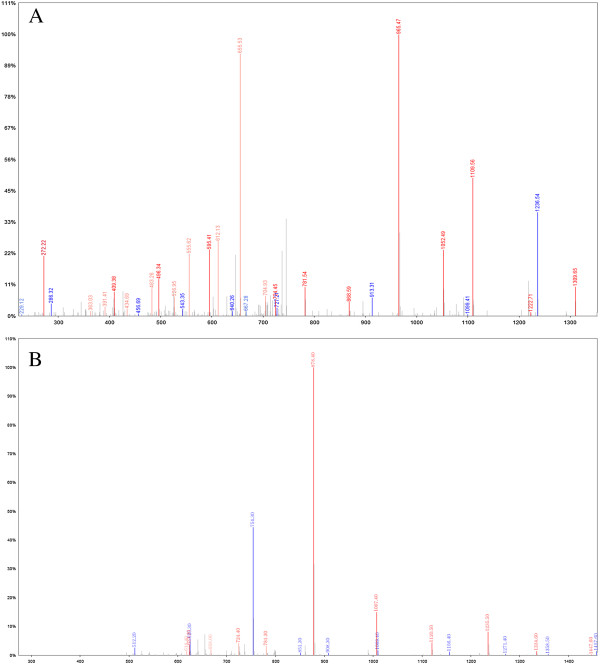
MS/MS identification of serum peptides as alpha-2-HS-glycoprotein precursor (A) and tubulin beta chain (B).

With this bead-based proteomic technology, two of the potential markers at m/z 1505 and 1618 Da could distinguish CRC from healthy volunteers, which is beneficial for further purification and identification with relatively high peak intensity. With this in mind, these proteins/peptides could be potential markers for further immunoassay trials. After fractionation by Nano Aquity UPLC (Waters Corporation, Milford, USA), the eluted plasma samples were further purified by C18 beads with 5 μm and 3.5 μm, then serially eluted with 5% and 95% acetonitrile. Samples were then subjected to LTQ Orbitrap XL MS/MS (Michrom Bioresources, Auburn, USA) analysis. The MS fingerprint was subjected to International Protein Index (IPI human v3.45 fasta with 71983 entries) searching for peptide sequence and further to NCBI database for protein identification. We had subsequently identified proteins by ion-spray mass spectrum. Mass spectrum with fragmentation pattern was identified through b and y ions which specify shown in Figure 6. Signal at m/z 1505 was identified as alpha-2-HS-glycoprotein precursor with the sequence G.VVSLGSPSGEVSHPR.K (IPI00022431.1 Gene_Symbol = AHSG, *P* = 1.08 × 10^−5^) and peak at m/z 1618 was identified as tubulin beta chain with R.AILVDLEPGTMDSVR.S sequence (IPI00011654.2 Gene_Symbol = TUBB, *P* = 4.10 × 10^−5^) (Figure 6 A and B).

## Discussion

The usefulness of multiple markers for diagnosis, prognosis and for predicting the risk of developing diseases or their complications is now widely recognized [13,28-30]. Various proteomic approaches have been applied to biomarker discovery using biological fluids.

Regarding the search of biomarkers in CRC patients, several studies have been reported. The use of patient tissue samples allows comparison of the tumor sample with adjacent non-involved tissue. Alteration of 18 proteins was identified during tumor formation [31]. Several other comparative proteomic studies of human CRC and adjacent normal tissue identified more proteins that appear to change consistently among patients with CRC [32-35]. Previously, our group identified gelsolin protein down-regulated in CRC [36]. However, sample sizes are frequently limiting as priority has to be given to the use of surgically obtained tissue for conventional diagnosis [7].

Proteomic profiling is based upon the fact that proteins represent the dynamic state of the cells, reflecting earlier pathological and physiological changes in the disease more accurately than genomic sequencing [37]. Proteomic patterns should assist in the detection of tumor biomarkers, as well as in evaluating the efficacy of anticancer drugs. The comparison of sera of cancer patients and healthy volunteers provided peaks from three differentially detected proteins, complement component 3a des-Arg, alpha-1-antitrypsin and transferrin, with 95% sensitivity and 91% specificity [38]. Another SELDI study identified protein peaks that could distinguish CRC patients from normal subjects and classify patients with tumors at different stages, with 95% sensitivity and specificity [39]. Our group identified protein peaks that could different lymph node positive CRC patients from negative ones [17]. There has also been an effort to identify putative biomarkers in urine samples of CRC patients, with 19 protein peaks showing different intensities from those of healthy individuals (78% sensitivity, 87% specificity) [40]. Further refinement and verification of these studies may eventually provide sets of clinically useful markers. Unfortunately, the proteome associated with CRC early diagnosis is currently poorly understood. Wang et al. identified two serum protein biomarkers (m/z 3961 and 5200 Da) for monitoring CRC [41]. Previously, an extensive proteomic analysis in the serum of patients with CRC was performed. A standardized serum preparation method for MALDI-TOF-MS was utilized based on WCX MB and was able to identify many valuable, low-abundance protein masses of interest [24]. However neither a characteristic protein cluster nor the structure identification of any of the proteins of the cluster was provided. Very recently it has been shown that clinical proteomic experiments can be still useful even when they deal with very small sample size [42].

In present study, a case control comparative analysis between CRC and health volunteers was performed by integrating the purification of proteins/peptides with WCX-MB, detection of peak intensity with MALDI-TOF MS, and profile analysis with ClinProt Tool software 2.2. Compared to controls, CRC shares 24 significantly differentiated proteins/peptides, including 9 up-regulated and 15 down-regulated peptides. By using the GA analysis, a cluster of 6 peptides at m/z 1208, 1467, 1505, 1618, 1656 and 4215 were developed as a classification mode, achieved a recognition capacity and a cross-validation of close to 100% to discriminate CRC from health volunteers. Also the classification mode could correctly classify CRC from health volunteers in blinded verification test, which surpass that of CEA.

The diagnostic capability of each peak at m/z 1208, 1467, 1505, 1618, 1656 and 4215 determined by ROC curve shows to be a highly accurate test (AUC> 0.88) [43]. These protein/peptide fragments with high specificity and sensitivity may be good serum biomarkers for CRC. Later studies in a larger population group are necessary to confirm this finding. Further evaluation identified the 1505 and 1618 Da marker as alpha-2-HS-glycoprotein precursor and tubulin beta chain, respectively by LTQ Obitrap XL.

Alpha-2-HS-glycoprotein precursor, a single chain form of alpha-2-HS-glycoprotein, is converged into the two chain form after the completion of carbohydrate side-chain processing [44]. Human serum protein alpha −2-HS glycoprotein is the human species homologue of bovine fetuin-A [45]. Alpha-2-HS-glycoprotein is a circulating plasma glycoprotein, produced abundantly during fetal development by multiple tissues, whereas in the adult, it is produced predominantly by the liver [46]. A number of studies suggest that alpha-2-HS-glycoprotein is a multifunctional protein [47]. Difference expression of alpha-2-HS-glycoprotein exists in breast cancer, CRC, lung cancer, liver cancer, head and neck cancer [48-54]. As for CRC, serum alpha-2-HS-glycoprotein inhibits tumor progression by blocking transforming growth factor-β1 (TGF-β1) binding to cell surface receptors, suppressing TGF-βsignal transduction, and inhibiting TGF-β-induced epithelial-mesenchymal transition [55]. Familial adenomatous polyposis (FAP) is one of the most important clinical hereditary forms of inherited susceptibility to CRC and is characterized by a high degree of phenotypic heterogeneity. Alpha −2-HS-glycoprotein was down-regulated in carpeting versus diffuse FAP patients and healthy donors, which was identified as serum molecule differently expressed in FAP patients [56]. Increasing chronological age is a risk factor for many types of cancer including colorectal. An understanding of the biology of aging and factors which regulate it may provide insight into cancer pathogenesis. Maxwell F et al. identified that decreasing alpha-2-HS-glycoprotein concentration was associated with increasing chronological age, which indicates accelerated biological aging. Furthermore, alpha-2-HS-glycoprotein levels can be used to distinguish between colon and rectal cancers [50]. However, serum alpha-2-HS-glycoprotein level was found not to be significantly changed by an ELISA test in CRC by another group [51]. Presently, up-regulated alpha-2-HS-glycoprotein precursor was determined in CRC, which may perform as a diagnosis marker for CRC.

Tubulin, the subunit protein of microtubules, has generally been thought to be exclusively a cytoplasmic protein in higher eukaryotes. It’s an important target for anti-tumor drugs [57]. Structurally, tubulin is an α/β heterodimer [58]. Abnormal expression of the specific β-tubulin isotype is related to resistance to chemotherapy in solid cancers [59-61]. This marker may assume a greater role in therapeutic decision making. However, overexpression of subtypes of β-tubulin, class III β-tubulin and class VI β-tubulin, is a purely prognostic factor related to biologic aggressiveness of CRC, surprisingly this phenomenon is restricted to female patients [62]. Early detection of malignancies of the gastrointestinal tract can lead to improved survival of patients worldwide. Beta-tubulin is a cancer-specific antigen in patients with CRC and other gastrointestinal malignancies, including gastric cancer, esophageal cancer and pancreatic cancer. Sensitivities ranged between 20% and 40% for various cancers with a specificity of 96% [63]. We identified that β-tubulin up-regulated in CRC, which show great potential for diagnosis for CRC.

In conclusion, this study is one of the few study to screen CRC related proteins/peptides in sera by combining WCX-MB and MALDI-TOF-MS according to our knowledge. The classification model we have set up have application in providing alternatives for CRC diagnosis, and the up-regulated alpha-2-HS-glycoprotein precursor and β-tubulin provide a better understanding of the pathogenesis in CRC or help in tailoring the use of chemotherapy to each patient, finally resulting in an improvement in patient outcome. Continuing research into the underlying pathophysiology of CRC will ultimately lead to more effective and better-tolerated therapies. Additional analysis of a larger set of individual samples in combination with more traditional immunoassays such as ELISA are required to further confirm whether high level of serum alpha-2-HS-glycoprotein precursor and β-tubulin increased odds ratios (ORs) of CRC in a nested case–control sample of CRC individuals such as those observed in this study.

## Competing interests

The authors stated that there are no conflicts of interest regarding the publication of this article.

## Authors’ contribution

NF carried out the mass spectrometry analysis, bioinformatics and statistical analysis, and drafted the manuscript. RK, XG and ML carried out the sample collection and purification. HC and YL carried out the identification of markers and CEA detection. CG participated in the design of the study. All authors read and approved the final manuscript.
